# Comparative Analysis of Gold Nanoparticle Synthesis Using PAMAM G2 Dendrimers via Microwave and Sonication Methods for Potential Cancer Theranostic Applications

**DOI:** 10.3390/molecules30234509

**Published:** 2025-11-22

**Authors:** Magdalena Grala, Bolesław Karwowski, Agnieszka Maria Kołodziejczyk

**Affiliations:** 1 Department of Chemical and Molecular Engineering, Faculty of Process and Environmental Engineering, Lodz University of Technology, Wolczanska 213, 93-005 Lodz, Poland; magdalena.grala@p.lodz.pl; 2 Food Science Department, Faculty of Pharmacy, Medical University of Lodz, Muszynskiego 1, 90-151 Lodz, Poland; boleslaw.karwowski@umed.lodz.pl; 3 Molecular and Nanostructural Biophysics Laboratory, Bionanopark Ltd., Dubois 114/116, 93-465 Lodz, Poland

**Keywords:** gold nanoparticles synthesis, PAMAM dendrimers of 2nd generation, XTT tetrazolium salt reduction tests, cellular viability

## Abstract

The rapid development of nanomedicine is driving extensive research and the synthesis of new nanomaterials. Biocompatible nanoparticles have the potential to serve as both imaging agents for medical diagnostics and carriers for targeted therapy. Among the various nanocomplexes investigated for cancer theranostics, gold nanoparticles stabilized by polyamidoamine (PAMAM) dendrimers have proven to be a promising platform. The unique physicochemical properties of gold nanoparticles, when combined with the branched architecture of PAMAM dendrimers, enhance stability, biocompatibility, and functionalization capability, enabling precise tumour targeting, improved imaging contrast, and controlled drug release. In this paper, we demonstrate the synthesis of gold nanoparticles stabilized by 2nd generation PAMAM dendrimers using three different methods: sonication, microwave, and unassisted techniques. The described synthesis approaches provide a rapid and straightforward method to achieve monodisperse particle size distribution and high colloidal stability up to 3 months. Physicochemical characterization of the nanocomplexes was carried out using ultraviolet-visible light spectroscopy, dynamic light scattering with zeta potential analysis, infrared spectroscopy, and atomic force microscopy. Furthermore, the effects of selected concentrations of PAMAM:HAuCl_4_ nanoparticles for all types of synthesis on human breast adenocarcinoma and human osteosarcoma cell lines were investigated using cytotoxicity assays. The results of the conducted tests show cytotoxicity values at a similar level. However, the sample synthesized using the sonication technique exhibited the lowest toxicity.

## 1. Introduction

Gold nanoparticles (AuNPs) stabilized by dendrimers have garnered extensive attention for their potential applications in cancer theranostics and therapy [1,2,3]. AuNPs are extensively studied and utilized in medicine [4] due to their excellent biocompatibility [5], chemical stability [6], cellular uptake ability [7], and modifiable surface properties [8]. The branched architecture of dendrimers, featuring multiple active amine groups, enables functionalization with a variety of polymers or antibodies, facilitating targeted therapeutic applications [9,10,11]. Dendrimer-stabilized gold nanoparticles represent a promising platform for drug delivery and gene therapy [12]. AuNPs stabilized by dendrimers have been investigated in particular as nanocarriers for doxorubicin, a widely used chemotherapeutic agent [13]. These complexes were also investigated for use in nanocapsules for the delivery of anticancer drugs that respond to glutathione or dithiothreitol [14]. In the future, AuNPs/PAMAM conjugates could be used to deliver other drugs. In this case, the drug (both, active substance and the nanocarrier) would undergo absorption, distribution, metabolism and excretion (ADME) processes [15]. When studying new drugs, it is crucial to identify design principles that minimize the potential for drug–drug interactions and reduce attritions, as well as to characterize the physicochemical properties that affect ADME. With the exception of oligonucleotides in gene therapy, each active substance must enter the cell or intercellular space, act, and then be metabolized and/or secreted [15].

AuNPs stabilized with PAMAM dendrimers can also be used as effective photosensitizers in photothermal and photodynamic cancer therapy. Saw et al. [16] have shown that PAMAM-based gold nanoparticle complexes display enhanced phototoxic effects relative to conventional photosensitizers bound to confeito-like gold structures. Integrating niosomes containing the chemotherapeutic agent paclitaxel and the photothermal therapy agent AuNPs/PAMAM yielded promising outcomes in the combined chemotherapy and photothermal treatment of breast cancer [17].

Due to the high atomic number of gold, AuNPs are excellent candidates for use as contrast agents. Combining them with PAMAM G5 dendrimers improves their performance in computed tomography (CT) imaging, facilitating the detection of tumours. This was examined on the human papilloma epithelial KB cell line [18]. AuNPs/PAMAM were also compared with a clinically used iodine-based contrast agent and exhibited improved X-ray attenuation properties [19]. Complexes of gold nanoparticles stabilized by PAMAM G5 dendrimers and additionally loaded with curcumin were developed for combined diagnostic and therapeutic applications [20]. These nanostructures were utilized in CT tumour imaging as contrast agents and simultaneously evaluated for their anticancer efficacy against colorectal adenocarcinoma. Modified AuNPs/PAMAM complexes were evaluated as dual-modality contrast agents for computed tomography and magnetic resonance imaging, with the aim of detecting lung cancer metastases with improved sensitivity and resolution [21].

One of the key challenges in the application of AuNPs/PAMAM complexes lies in ensuring their stability while enabling a facile and rapid synthesis process. Various environmental factors, including temperature [22], pH [23], and ionic strength [24], can significantly impact the colloidal and chemical stability of these nanostructures. The long-term stability and appropriate storage conditions of dendrimer-based nanoparticles remain active areas of investigation [25,26].

Another aspect of investigating AuNPs/PAMAM is selecting the dendrimer generation. Most research is focused on the 5th generation of PAMAM [17,18,27] due to its high number of functional groups (128), suitable molecular size for gold ion encapsulation, and its ability to stabilize gold nanoparticles while reducing aggregation. AuNPs synthesized using higher generations of PAMAM dendrimers (e.g., G5 or G6) demonstrate reduced polydispersity and enhanced colloidal stability under identical synthetic conditions. This is due to the greater number of coordination sites increases the extent of nucleation and stabilization [28]. Additionally, toxicological studies have shown that higher generations of PAMAM dendrimers tend to exhibit increased cytotoxicity compared to lower generations [29,30]. Furthermore, due to their cost-efficient synthesis process, low-generation dendrimers are a more economically viable and practical alternative to high-generation ones for biomedical applications.

**Figure 1:**
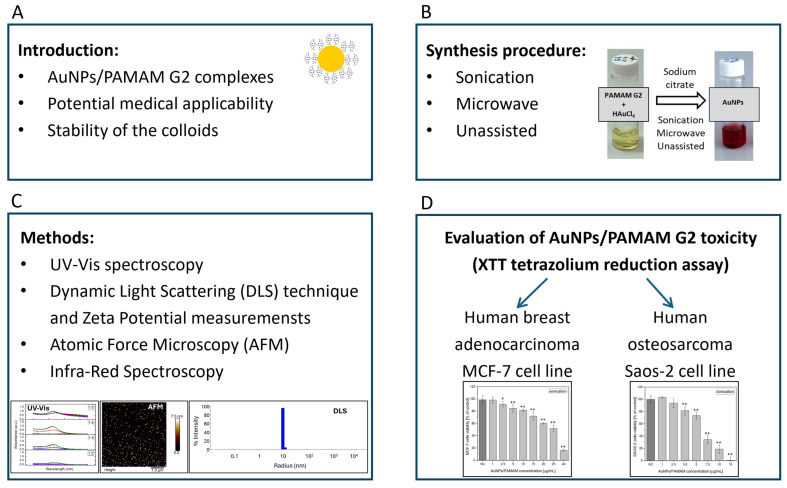
The schematically illustrated sections of the manuscript including the introduction (**A**), synthesis procedure (**B**), methods and results (**C**) and cytotoxicity evaluation on human breast adenocarcinoma MCF-7 cell line and human osteosarcoma Saos-2 cell line (**D**). Statistical significance of cells viability versus respective negative control (**D**) is indicated when appropriate (* *p* < 0.01, ** *p* < 0.001).

In this context, we aimed to develop a rapid and straightforward method for synthesizing gold nanoparticles using 2nd generation of PAMAM dendrimers. Our goal was to achieve a monodisperse particle size distribution and high colloidal stability. Figure 1 illustrates the schematic representation of the manuscript sections including synthesis procedure (B), methods and results (C) and cellular viability evaluation on cancer cells (D).

The synthesis was carried out using sonication or microwave methods, as well as without applying these techniques for comparison purposes. Sodium citrate was employed as a reducing agent. This stabilizer is widely used in nanoparticle synthesis due to its effective capping ability. The stability of the colloids was investigated over a period of 3 months. The nanoparticles were characterized using the following techniques: ultraviolet-visible spectroscopy (UV-Vis), dynamic light scattering (DLS), atomic force microscopy (AFM) and Fourier transform infrared spectroscopy (FTIR). We evaluated the effects of the selected gold nanoparticles on two cancer cell lines: MCF-7 (human breast adenocarcinoma) and Saos-2 (human osteosarcoma). Cytotoxicity was assessed using a 2,3-bis[2-methoxy-4-nitro-5-sulfophenyl]-2H-tetrazolium-5-carboxanilide (XTT tetrazolium) reduction assay.

## 2. Results

### 2.1. Synthesis Reaction of AuNPs/PAMAM G2 Complexes

**Figure 2:**
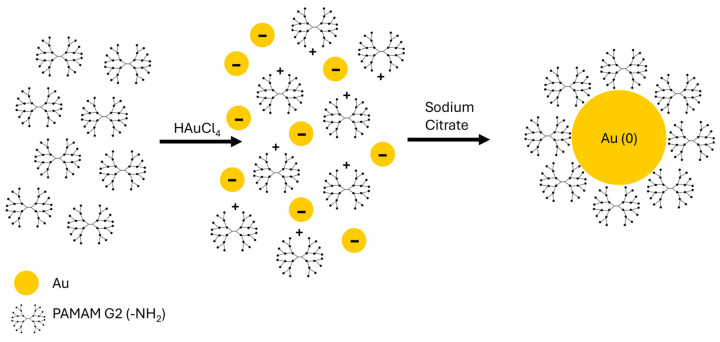
Schematic representation of AuNPs/PAMAM G2 synthesis. Gold (III) chloride hydrate (HAuCl_4_·3H_2_O) precursor and ethylenediamine core polyamidoamine (PAMAM) dendrimers of the 2nd generation with 16 functional primary amino groups on the surface are mixed in aqueous solution. Then, sodium citrate was added to reduce the Au(III) centres to Au(0) metallic gold.

The AuNPs can be easily obtained by mixing a gold salt such as HAuCl_4_ with a dendrimer in aqueous solution. Molar ratios of PAMAM to HAuCl_4_ were selected over a wide range, from 1:0.5 to 1:6. Negatively charged gold precursor ions (AuCl_4_^−^) are attracted to and concentrated by the numerous protonated amine groups (-$NH_{3}^{+}$) on the surface and within the interior of the dendrimer via electrostatic interactions [31]. Upon addition of sodium citrate, the reduction process begins: Au(III) ions are converted into neutral gold atoms (Au(0)), which subsequently nucleate and grow into nanoparticles. The pre-concentration of the gold ions by the dendrimer facilitates the formation of uniform AuNPs. After the metallic core has formed, the PAMAM molecules coat the nanoparticle’s surface. Figure 2 illustrates the schematic synthesis reaction of AuNPs/PAMAM G2 complexes. This structure is typical of dendrimer-stabilized nanoparticles and is characteristic of low-generation PAMAM dendrimers [32]. The primary mechanism for direct chemical linkage involves the persistent coordination bond between the surface gold atoms of the AuNP and the nitrogen atoms from the dendrimer’s amine groups (-NH_2_ and -NH or -N). These surface coordination bonds are crucial for capping and preventing immediate aggregation. However, long-term stability is ensured by non-covalent forces: the branched architecture of the dendrimers provides steric stabilization, while the terminal amine groups contribute to electrostatic stabilization [33].

### 2.2. Physico-Chemical Properties of AuNPs/PAMAM G2

**Figure 3:**
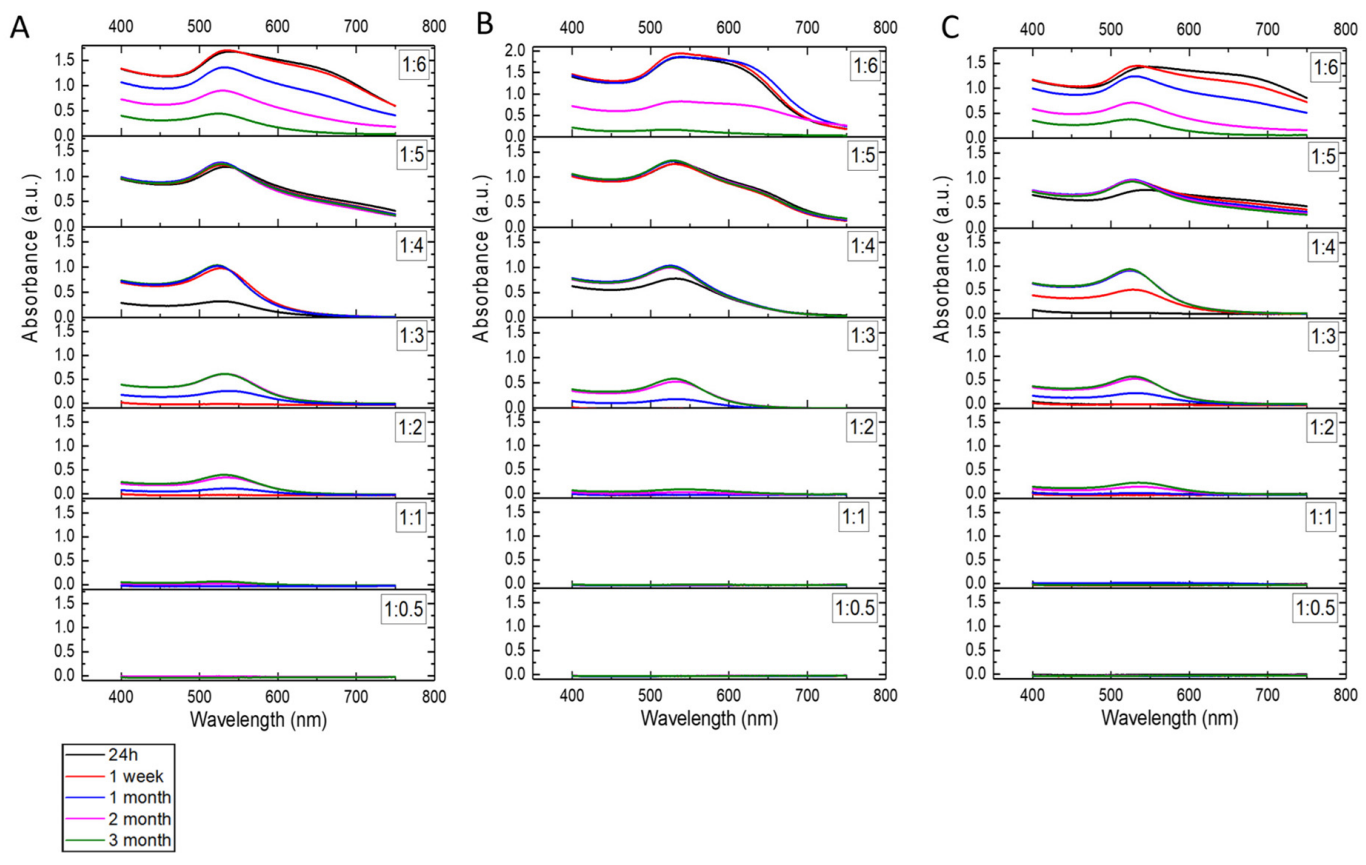
UV-Vis spectra of Au/PAMAM complexes. NPs were synthesized using sonication (**A**), microwaves (**B**), and unassisted (**C**) methods. The different PAMAM G2:HAuCl_4_ concentration ratios, ranging from 1:0.5 to 1:6, are indicated on the spectra. The black curves represent spectra measured 24 h after synthesis, the red curves—1 week, the blue curves—1 month, the pink curves—2 months, and the green curves—3 months after synthesis.

The formation of gold nanoparticles was confirmed by UV-Vis spectroscopy. The spectra were collected for all samples (Figure 3) after 24 h, 1 week, 1 month, 2 months, and 3 months. For the samples with a PAMAM G2:HAuCl_4_ concentration ratio of 1:0.5 and 1:1, no absorbance peaks were observed, indicating that no nanoparticles were formed. These samples were not subjected to further testing. The AuNPs/PAMAM samples exhibited an absorption band in the range of 525–550 nm. Additionally, a broader shoulder was observed in the 600–750 nm range for the 1:5 and 1:6 samples. To facilitate comparison, the absorbance scale is consistent for most samples, except for the 1:6 samples shown in Figure 3B. As expected, the absorbance increases with increasing HAuCl_4_ concentration. The most stable colloids with high absorbance intensities appear to be the 1:4 and 1:5 samples, with absorbance peaks at 526 nm (1:4 sonication), 532 nm (1:4 microwaves), 528 nm (1:4 unassisted), 534 nm (1:5 sonication), 538 nm (1:5 microwaves), and 544 nm (1:5 unassisted).

The hydrodynamic diameter values and mass percentages for AuNPs/PAMAM G2 colloids from 1:2 to 1:5 are listed in Table 1, Table 2 and Table 3 for the sonication, microwave, and unassisted methods, respectively. At lower concentrations of chloroauric acid, namely 2 and 3 mM (samples labelled as 1:2 and 1:3), measurable nanoparticles are not formed immediately (for syntheses using microwave and unassisted techniques). The measurements were not possible at that point in time due to a high sum of squares (SOS) error and poor autocorrelation function fitting. Additionally, no gold nanoparticles were detected after 1 week for sample 1:2, when analyzed using the microwave and unassisted methods. This may result from the strong complexation of Au(III) ions by the dendrimer’s amino groups and the slow kinetics of Au(III) reduction by sodium citrate in the presence of chloride ions. Initially this process leads to the formation of small clusters or complexes, followed by slow growth and aggregation. In the case of sonication-assisted synthesis, the elevated temperature further accelerates the reaction kinetics; however, at low chloroauric acid concentrations, stable colloids were not obtained (they change over time). At a higher concentration of chloroauric acid (6 mM), corresponding to the sample labelled 1:6, precipitation of material and formation of an unstable colloid are observed. The 1:6 samples from all synthesis methods were unmeasurable by the DLS technique, most likely due to the formation of large agglomerates and aggregates. At a chloroauric acid concentration of 5 mM (colloid labelled as 1:5), an excess of Au is likely present, which promotes aggregation and the formation of larger and polydisperse nanoparticles. The optimal concentration appears to be 4 mM chloroauric acid (sample labelled as 1:4), which, under elevated temperature conditions during sonication-assisted synthesis (80 °C for 60 min), results in the immediate formation of monodisperse nanoparticles with a diameter of approximately 19 nm that remain stable up to 3 months. In the case of unassisted synthesis, where no temperature increase is applied and nucleation proceeds much more slowly, monodisperse nanoparticles are obtained after one week, with a diameter of 16 nm. For microwave-assisted synthesis, where nucleation occurs very rapidly (within 45 s), two distinct nanoparticle populations were formed immediately, with diameters of approximately 9 nm and 100 nm, both of which remain stable for a period of 3 months. The samples with the most repeatable and stable results, as determined by UV-Vis and DLS measurements, were synthesized with a PAMAM G2:HAuCl_4_ concentration ratio of 1:4. Therefore, these samples were selected for cell viability studies.

**Figure 4:**
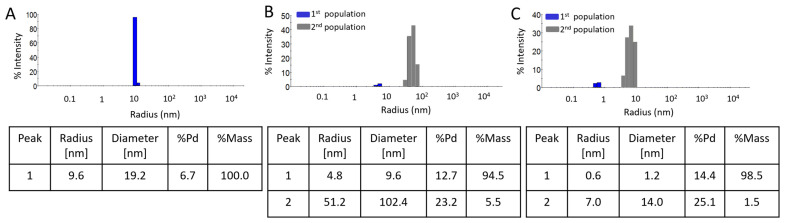
The examples of DLS size distribution of AuNPs/PAMAM at 1:4 PAMAM G2:HAuCl_4_ concentration ratios 24 h after synthesis for sonication (**A**), microwaves (**B**) and unassisted (**C**) methods obtained from the DLS technique. The blue and grey bars represent the 1st and 2nd populations, respectively.

Figure 4 illustrates the apparent hydrodynamic distribution of the selected samples exhibiting the highest repeatable and stability with PAMAM G2:HAuCl_4_ concentration ratio of 1:4 using three different synthesis methods including sonication (A), microwaves (B) and unassisted (C). After sonication synthesis, a monodisperse population of gold nanoparticles with a diameter of 19.2 nm and a polydispersity 6.7% was obtained. The microwave method resulted in polydisperse colloids with two populations of particles measuring 9.6 and 102.4 nm in radius with polydispersity indices of 14.7% and 25.2%, respectively. The unassisted synthesis leads to the formation of two particle populations with radii of 1.2 and 14 nm (with polydispersity of 14.4% and 25.1%, respectively). The first population may correspond to unreacted synthesis products resulting from the continued reduction of gold ions over time, while the second population corresponds to the size of gold nanoparticles.

**Figure 5:**
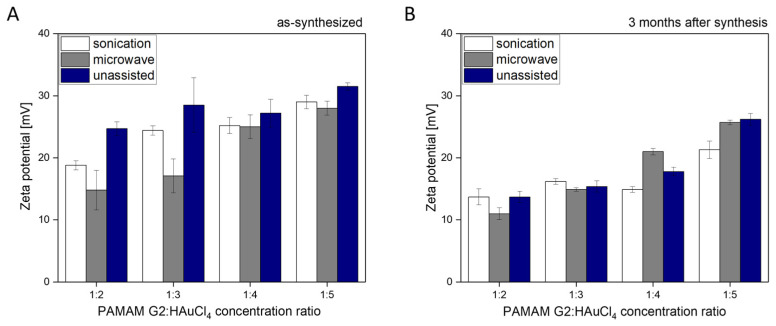
Zeta potential values for AuNPs/PAMAM G2 after synthesis (**A**) and 3 months post-synthesis (**B**).

Zeta potential study yielded gold concentration-dependent values for the AuNPs/PAMAM colloid samples (Figure 5A). The zeta potential values ranged from 15 to 30 mV. It is worth noting that immediately after unassisted synthesis, the zeta potential values for colloids 1:2, 1:3, 1:4, and 1:5 are similar, ranging from 25 to 29 mV. In the unassisted method, no temperature increase is applied, and nucleation proceeds much more slowly. After three months, a decrease in zeta potential of approximately 5 to 10 mV was observed for nearly all samples (Figure 5B). This observation is evident for colloids synthesized using sonication and unassisted techniques. This observation indicates modification of the surface of AuNPs/PAMAM G2 complexes during long-term storage. There is no significant change in zeta potential values of microwave-synthesized samples between the initial measurement and after three months. The decrease in zeta potential of the synthesized colloids after 3 months has led us to hypothesize that residual cationic dendrimer groups are being neutralized by residual citrate ions in these colloids, as described in the Section 3.

**Figure 6:**
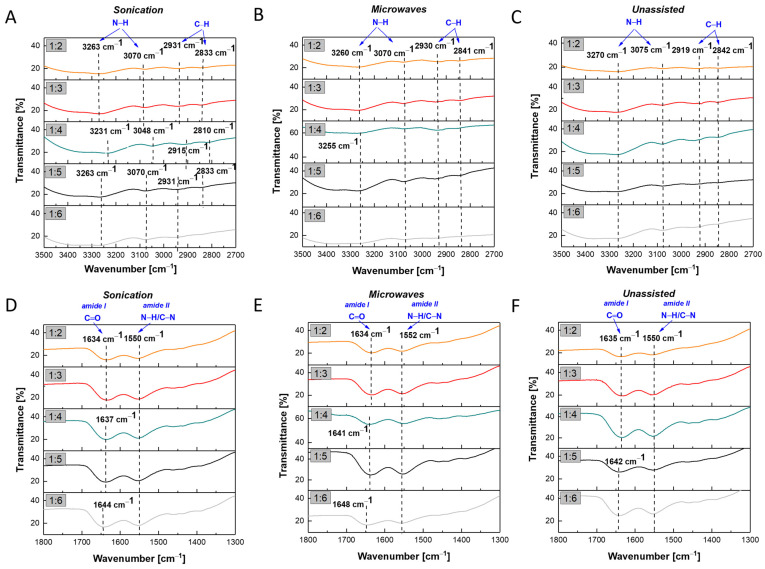
FTIR spectra of AuNPs/PAMAM G2 with different PAMAM G2:HAuCl_4_ concentration ratios of analyzed ranges: 3500–2700 cm^−1^ (**A**–**C**) and 1800–1300 cm^−1^ (**D**–**F**).

For the chemical characterization of AuNPs/PAMAM G2, infrared spectroscopy was performed. All characteristic FTIR peaks are marked in Figure 6. For better visualization, two spectral regions were magnified: 3500–2700 cm^−1^ (Figure 6, upper panel) and 1800–1300 cm^−1^ (Figure 6, lower panel). To improve clarity, the spectra were separated, and characteristic peaks and shifts were marked individually for each plot. The FTIR spectra showed broad vibration peaks with maxima ranging from 3270 cm^−1^ to 3230 cm^−1^. These peaks, around 3250 cm^−1^, are related to the N–H stretching vibration originating from PAMAM association [34]. Another broad vibration peak appeared at approximately 3070 cm^−1^ (Figure 6A,B), except for the 1:4 sonication sample–3048 cm^−1^ (Figure 6A), and 3075 cm^−1^ (Figure 6C). These bands correspond to N–H bending vibrations. Additionally, the peaks around 2930 cm^−1^ and 2840 cm^−1^ indicate unsymmetrical and symmetrical stretching vibrations of methylene groups, respectively [34]. The bands shown in the lower panel of Figure 6, at approximately 1640 cm^−1^ and 1550 cm^−1^, can be assigned to amide I (C=O stretching) and amide II (N–H deformation and C–N stretching) vibrations, respectively, which are characteristic of dendrimer branches [35]. Shifts in the N–H stretching vibration bands within the range of 3500–2700 cm^−1^ toward lower wavenumbers were observed for all samples. In the case of the sonication synthesis, characteristic peaks appeared at 3263 cm^−1^ (samples 1:2, 1:3, 1:5, and 1:6) and 3231 cm^−1^ (sample 1:4). For the microwave-assisted synthesis, the corresponding peaks were detected at 3260 cm^−1^ (samples 1:2 and 1:3) and 3255 cm^−1^ (samples from 1:4 to 1:6). The unassisted synthesis yielded a single peak at 3270 cm^−1^ for all samples. The observed red shifts indicate interactions between Au ions and the amine groups of PAMAM G2, leading to changes in hydrogen bonding and/or interactions between the dendrimer and the gold surface.

In the Section 3, we proposed a hypothesis regarding the formation of AuNPs/PAMAM G2 complexes. For the 1:4 sample, the number of Au ions appears optimal for randomly occupying the most energetically favourable binding sites on PAMAM G2 dendrimers, which may also contribute to the observed shifts in the N–H bending vibrations.

**Figure 7:**
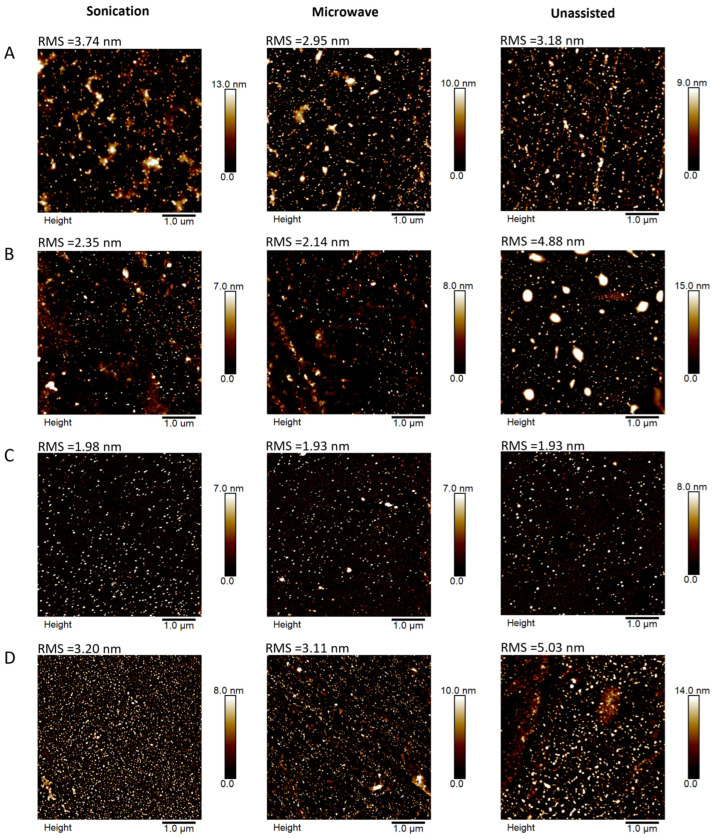
Surface topography images of AuNPs/PAMAM G2 synthesized using sonication (left column), microwave (middle column) and unassisted (right column) methods, and deposited on silicon substrates marked 1:2 (**A**), 1:3 (**B**), 1:4 (**C**) and 1:5 (**D**), respectively. Scan size 5 × 5 µm at a resolution of 512 × 512 data points.

The AFM images (Figure 7) of AuNPs/PAMAM samples deposited on silicon substrates show the surface topography for a scan size of 5 × 5 µm. The images of the 1:2 and 1:3 samples reveal nanoparticles and some larger structures, which could be aggregates or agglomerates. For the 1:4 and 1:5 samples, well-dispersed nanoparticles can be observed; only the 1:5 unassisted sample demonstrates larger nanostructures. The root mean square (RMS) value reaches a minimum for all 1:4 samples—below 2 nm. This indicates that the morphology of these samples is relatively homogeneous.

### 2.3. Investigation of AuNPs/PAMAM G2 Cytotoxicity on MCF-7 and Saos-2 Cell Lines

**Figure 8:**
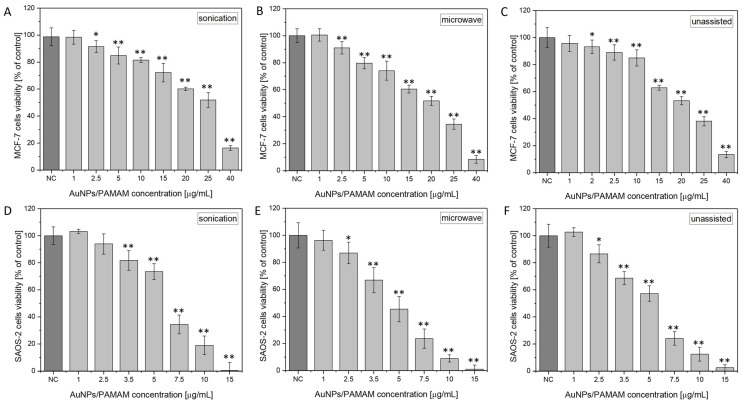
The viability of MCF-7 (**A**–**C**) and Saos-2 (**D**–**F**) cells investigated by the XTT test after incubation with AuNPs/PAMAM G2. NC refers to negative control (i.e., untreated cells). Statistical significance versus respective negative control is indicated when appropriate (* *p* < 0.01, ** *p* < 0.001).

Figure 8 presents the viability of the Saos-2 and MCF-7 cell lines after exposure to selected 1:4 AuNPs/PAMAM G2 samples. The analysis includes a comparison of cell viability obtained using three synthesis methods: sonication, microwave, and unassisted. According to the obtained results, AuNPs/PAMAM G2 caused a decrease in the viability of cells in a dose-dependent manner and induced a cytotoxic effect above the 2.5 and 3.5 µg/mL concentrations for MCF-7 and Saos-2 cell lines, respectively. The obtained results were confirmed by statistical tests (ANOVA, (* *p* < 0.01, ** *p* < 0.001). Table 4 and Table 5 show the EC_10_, EC_25_, and EC_50_ (effective concentration) values correspond to the 90, 75, and 50% of the control cellular viability and their standard deviations for MCF-7 and Saos-2, respectively. According to the results, the lowest EC values were observed for the Saos-2 cell line across all synthesis methods. Compared with MCF-7, the EC_10_ value for Saos-2 was approximately two times lower, while the EC_25_ and EC_50_ values were about three and four times lower, respectively. It is noteworthy that AuNPs/PAMAM G2 nanoparticles synthesized via the sonication method exhibited the lowest cytotoxicity toward both MCF-7 and Saos-2 cell lines. This synthesis yielded a monodisperse nanoparticle population with an average diameter of approximately 19 nm (Table 1). In contrast, nanoparticles obtained using the unassisted method were smaller, with an average diameter of around 15 nm (Table 3). The microwave-assisted synthesis resulted in the formation of two distinct nanoparticle populations, with 95% of the mass comprising gold nanoparticles of 9.6 nm in diameter (Table 2). These findings confirm that smaller nanoparticles are associated with increased cytotoxicity. Importantly, the zeta potentials of the colloidal AuNPs/PAMAM G2 systems selected for cytotoxicity testing—prepared using all three synthesis methods: sonication, microwave-assisted, and unassisted—were comparable within the margin of error, measuring 25.2, 25.0, and 27.2 mV (Figure 5), respectively. Therefore, the observed differences in cytotoxicity can be attributed primarily to nanoparticle size rather than surface charge.

## 3. Discussion

The interaction between nanoparticles and human cells plays a crucial role in determining the biological response to nanomaterials. Particles with diameters below 200 nm tend to be more efficiently taken up by a wide range of human cell types [36]. Furthermore, introducing positively or negatively charged functional groups of polymers, amino acids, or peptides/proteins can significantly enhance nanoparticle uptake [37]. Understanding the factors that influence cellular uptake, such as size, surface properties, cell type and endocytotic pathways, enables more effective labelling and selection of cells and nanoparticles for use in in vitro and in vivo applications [38]. An important question regarding the interaction between nanoparticles and cells is whether this interaction involves direct absorption or triggers a cascade of reactions involving specific receptors on the cell membrane. However, once nanoparticles have entered cells, the mechanisms responsible for their excretion may also be activated. In this study, we present a new synthesis method for gold nanoparticles stabilized with second-generation PAMAM dendrimers. The choice of dendrimers was driven by their unique properties including their outstanding host-guest chemistry, multivalent geometry, high water solubility, excellent encapsulation efficiency, and the adaptive surface architecture and their proven effectiveness as drug solubilizing agents [39].

The process of gold nanoparticle stabilization using PAMAM dendrimers depends on the dendrimer generation (and thus on their size). Garcia et al. [35] found that low-generation PAMAM dendrimers form AuNP complexes coated with a coordination shell of dendrimers. In the case of low-generation dendrimers (below G3), AuNPs initially form on the surface of PAMAM dendrimers and are subsequently stabilized through interactions with multiple dendrimer molecules. For PAMAM G4 and higher generations, the structure features a greater number of branches, which provide internal voids for complex formation and offer effective protection. In the study by Avila-Salas [40], molecular dynamics simulations were conducted to investigate the stabilization mechanism of AuNPs using PAMAM G0 and PAMAM G4 dendrimers. The results showed that PAMAM G0 tends to surround AuNPs, forming a so-called stabilization sphere around the dendrimers. In contrast, the PAMAM G4 dendrimer encapsulates AuNPs, providing stabilization sites through its internal cavities. Due to its larger size and higher branching density, it can stabilize more than one metallic system within its internal pockets.

A thorough physicochemical analysis of the synthesized nanoparticles was performed. The typical band of UV-Vis spectra corresponding to surface plasmon resonance (SPR) in AuNPs is typically observed in the range of 500–550 nm [41]. In the present study, the AuNPs/PAMAM samples clearly exhibit SPR bands in the range of 525–550 nm. The UV-Vis measurements confirm the formation of AuNPs in all samples except for those with a PAMAM G2:HAuCl_4_ ratios of 1:0.5 and 1:1. A second, broader shoulder observed in the 600–750 nm regions in some samples (1:5 and 1:6) suggests strong interactions between nanoparticles within aggregates [42]. As the PAMAM:HAuCl_4_ ratio increases, the SPR band shifts slightly towards longer wavelengths, indicating the formation of larger gold nanoparticles [43]. The stability of AuNPs/PAMAM G2 colloids was monitored over a three-month period. In the case of the 1:6 samples, a decrease in absorbance intensity over time was observed, indicating nanoparticle loss due to precipitation and aggregation [44]. In contrast, the 1:2, 1:3, and 1:4 samples showed an initial increase in absorbance intensity, which then remained approximately constant over time. This increase is attributed to the continued reduction of gold ions, resulting in a higher concentration of AuNPs [45]. Furthermore, the higher absorbance observed in samples with increased HAuCl_4_ concentration indicates a greater concentration of nanoparticles in the colloids.

Time-stability studies were also conducted using dynamic light scattering (Table 1, Table 2 and Table 3). The measured hydrodynamic radius of samples 1:4 and 1:5, across all synthesis methods, remained mostly stable for up to 3 months, although the samples were polydisperse. In contrast, nanoparticles in samples 1:2 and 1:3 synthesized by microwave and unassisted methods were detectable only after one week or one month. This observation is also reflected in the UV–Vis spectra, where an increase in absorbance over time was noted. The synthesis reaction may have remained incomplete after 24 h due to slow reaction kinetics, subsequently proceeding spontaneously.

Nanoparticle stability is typically associated with zeta potential values of approximately ±30 mV or higher [46]. As shown in Figure 5, samples 1:4 and 1:5 reached values close to 30 mV, indicating that these formulations are among the most stable. However, the literature also reports examples of nanoparticles with zeta potential values around −20 mV exhibiting high long-term stability [47]. Additionally, the decreased zeta potential values after 3 months (Figure 5) suggest temporal changes in the functional groups of PAMAM G2 dendrimers, which is in good agreement with our previous manuscript [25]. Continuing, it suggests a hypothesis relating to surface modification of the synthesized colloids, which may result from the neutralization of residual cationic dendrimer groups by residual citrate ions. Based on the DLS results, we exclude that the changes in the zeta potential of AuNPs/PAMAM G2 after 3 months are related to their decomposition.

Infrared spectral analysis was conducted to assess the chemical composition of the AuNPs/PAMAM complexes (Figure 6). The obtained spectra revealed characteristic bands corresponding to N–H, O–H, and amide groups, consistent with the literature [34,35]. However, a red shift was observed compared to the reference FTIR spectrum of PAMAM G2 [48], most notably in the 1:4 sonication sample, within the range of 3500 cm^−1^ to 2700 cm^−1^. This red shift in the N–H stretching vibration may indicate the formation of hydrogen bonds and/or coordination interactions between the dendrimer and the gold surface [49,50]. These interactions likely contribute to enhanced colloidal stability by preventing nanoparticle aggregation in solution. For the remaining samples, the band positions are very similar. The physicochemical characterization of the synthesized nanoparticles allowed the selection of the most reproducible and stable AuNPs/PAMAM complexes for subsequent cell viability studies.

An additional noteworthy aspect is the influence of the PAMAM G2:HAuCl_4_ molar ratio on nanoparticle formation. This molar ratio was systematically varied across a broad range, from 1:0.5 to 1:6. Below, we present a hypothesis regarding the formation of AuNPs/PAMAM G2 complexes as a function of the synthesis method. The synthesis was initiated using a 0.5 mM concentration of HAuCl_4_, with the goal of minimizing reagent consumption and thereby reducing process costs. However, for systems with 1:0.5 and 1:1 ratios, the HAuCl_4_ concentration proved too low compared to the dendrimer concentration. This likely resulted in strong stabilization of Au ions by PAMAM G2, which in turn prevented the reduction of gold to Au(0) and the initiation of the nucleation process. For molar ratios of 1:2 and 1:3, no measurable nanoparticles were formed immediately after synthesis, regardless of whether microwave-assisted or unassisted methods were employed. Furthermore, after one week, no gold nanoparticles were detected in the 1:2 sample synthesized using either approach. This phenomenon may be attributed to the strong complexation of Au(III) ions by the amine groups of the dendrimer, whereby gold ions randomly occupy the most reactive sites of PAMAM G2 dendrimers. Additionally, the slow kinetics of Au(III) reduction by sodium citrate in the presence of chloride ions may further hinder nanoparticle formation. In this manner, small clusters or complexes are initially formed, followed by gradual growth and aggregation. Elevating the temperature to 80 °C (via sonication) accelerates the reaction kinetics, enabling the formation of gold nanoparticles even at PAMAM G2:HAuCl_4_ molar ratios of 1:2 and 1:3; however, these nanoparticles undergo temporal instability. For the sonication method, a PAMAM G2:HAuCl_4_ molar ratio of 1:4 appears optimal, yielding size-stable gold nanoparticles that remain stable for up to three months. This may be attributed to the increased level of gold ions relative to the limited number of energetically favourable binding sites of dendrimers. In the case of microwave-assisted synthesis (sample 1:4), where nucleation occurs rapidly, two distinct nanoparticle populations form immediately and also exhibit long-term stability over three months. In contrast, for unassisted synthesis at room temperature (sample 1:4), nucleation proceeds significantly more slowly, and monodisperse nanoparticles are obtained only after one week. At molar ratios of 1:5 and 1:6, all energetically and spatially accessible sites are saturated with gold ions, leading to material precipitation, which promotes aggregation and the formation of larger, polydisperse nanoparticles.

The cytotoxicity of AuNPs in cancer cells depends on their size, dose, shape, surface chemical radicals, and charge [51,52,53,54]. Even when both AuNPs are the same size, utilized in the same cell lines, and present at the same concentration, Goodman et al. [55] have shown that positively charged AuNPs are more cytotoxic than negatively charged AuNPs. The affinity between the nanoparticles’ positive charge and the cell membrane’s negative charge is what causes this action [56,57]. The affinity between the nanoparticles’ positive charge and the cell membrane’s negative charge is the mechanism that generates this action [58]. In our study, we selected breast carcinoma and osteosarcoma cells for cellular viability tests. According to statistical data, breast cancer is one of the most prevalent types of cancer [59]. One of the applications of nanoparticles is their use in theranostics, which is a field that combines the principles of therapy and diagnostics [60]. To evaluate the potential of AuNPs/PAMAM complexes in biological applications, the most stable colloid with a 1:4 PAMAM G2:HAuCl_4_ concentration ratio was selected for cytotoxicity testing. The XTT test results indicate that the sonication method produces the least toxic nanoparticles, as demonstrated in Figure 8. By comparing the toxicity results for MCF-7 cells obtained from the microwave synthesis using different stabilizers—PAMAM G2 (Figure 8B) and PAMAM G4 [24]—we can conclude that both types of nanocomplexes disclose similar levels of toxicity. The EC_10_, EC_25_, and EC_50_ values obtained in the presented work are 3.3, 9.3, and 20 μg/mL (see Table 4), respectively. In our previous work [25], we obtained values of 10, 15, and 24.5 μg/mL. Initially, we hypothesized that the nanocomplexes stabilized with PAMAM G2 would exhibit lower cytotoxicity, based on literature reports indicating that lower-generation PAMAM dendrimers tend to be less toxic [30]. However, the diameter of the AuNPs stabilized with PAMAM G4 was approximately 20 nm, while those stabilized with PAMAM G2 exhibited two distinct populations of particles, approximately 10 nm and 102 nm in diameter. Smaller nanoparticles are generally known to display higher cellular uptake and, consequently, greater cytotoxic effects due to their enhanced interactions with cellular membranes [61]. When comparing the impact of nanoparticles synthesized from PAMAM G2 with those from PAMAM G4, it should be noted that they consist of two separate populations with significantly different hydrodynamic diameters. Therefore, the size differences among the nanoparticles could play a crucial role in determining cell viability.

Despite the growing interest in nanostructures within medicine and pharmacy, numerous challenges still hinder the clinical application of this nanotechnology. Younis et al. [62] identified key factors that should be considered in nanomedicine-related research to ensure clinical translatability. These factors can be categorized into five main areas: rational design during the research and development phase, recruitment of representative preclinical models, careful planning of clinical trials, development of specific and harmonized regulatory protocols, and consideration of non-traditional funding strategies [62]. Before nanoparticle-based products can be applied, several translational barriers must be addressed [63,64]. These include the complexity of large-scale manufacturing, safety and toxicity concerns arising from unknown long-term effects and accumulation potential, as well as significant challenges posed by biological barriers such as the blood–brain barrier and the body’s immune responses.

In the future, in vivo studies on the biocompatibility of AuNPs/PAMAM G2 will pose a significant challenge for researchers. Their application in medicine still requires a series of investigations, including clinical trials. The potential sites of nanoparticle accumulation in the human body—such as the circulatory system—suggest that these conjugates should also be examined in so-called barrier systems, including the blood–brain barrier, endothelium, intestine, and others. It is also important to address whether these nanoparticles may accumulate in vital organs, induce oxidative stress, or activate the immune system. Another aspect of their potential commercial use is large-scale synthesis. Sonication and unassisted methods can be successfully scaled up, whereas microwave-assisted synthesis is more difficult to scale, although it offers the advantage of a very short reaction time.

## 4. Materials and Methods

### 4.1. Synthesis of AuNPs/PAMAM G2

Gold (III) chloride hydrate (HAuCl_4_·3H_2_O) and ethylenediamine core polyamidoamine (PAMAM) dendrimers of the 2nd generation with 16 functional primary amino groups on the surface (20% methanol solution) (PAMAM G2) were purchased from Sigma-Aldrich (St. Louis, MO, USA), while sodium citrate was purchased from Chempur (Piekary Śląskie, Poland). The synthesis of gold nanoparticles and PAMAM dendrimer complexes was previously described [40,65].

**Figure 9:**
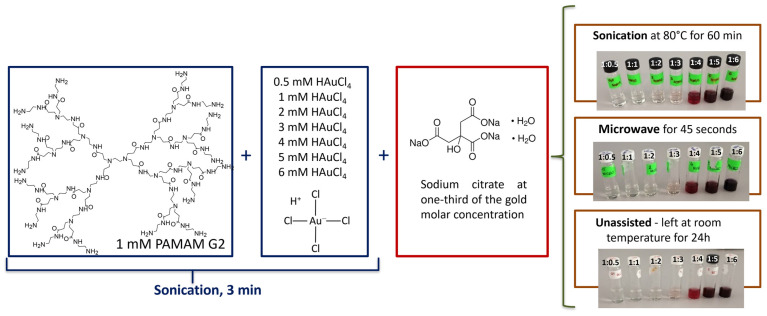
The scheme of AuNPs/PAMAM G2 procedure synthesis. First, chloroauric acid was sonicated with PAMAM G2 dendrimers for three minutes. Next, sodium citrate was added to the solution, which then underwent further stages of sonication or microwave treatment. In parallel, colloids were prepared using the unassisted method, involving vortexing the mixture and leaving it to react for 24 h.

In this study, AuNPs/PAMAM G2 complexes were prepared using different methods: sonication, microwave, and without sonication or microwave (unassisted synthesis), with sodium citrate as reducing agents. 1 mL of 1 mM PAMAM G2 aqueous solution was mixed with 1 mL of 0.5 mM, 1 mM, 2 mM, 3 mM, 4 mM, 5 mM, and 6 mM HAuCl_4_ solutions (labelled sample 1:0.5, 1:1, 1:2, 1:3, 1:4, 1:5 and 1:6, respectively) and sonicated for 3 min at room temperature. Then, 1 mL of sodium citrate was added at one-third of the gold molar concentration in the chloroauric acid mixture to reduce the Au complex. In the next step, one part of the samples was sonicated at 80 °C with a power output of 180 W for 60 min, a second part was microwave-treated at 800 W for 45 s, and a third part was left at room temperature (without sonication or microwaves) for unassisted synthesis. Most of the obtained colloids changed colour to red, indicating the formation of gold nanoparticles. The scheme of the synthesis procedure was presented in Figure 9. DLS and UV-Vis measurements were conducted on the initial concentrations obtained directly after synthesis, without prior dilution. The AuNPs/PAMAM G2 colloids were centrifuged at 12,000× *g* for 30 min, which allowed for the removal of extraneous ions and byproducts of redox processes. This procedure is consistent with literature reports [66,67]. The resulting pellet was washed twice and resuspended in high-grade quality water.

### 4.2. Ultraviolet-Visible Spectroscopy

The UV-Vis absorption spectra of AuNPs/PAMAM were measured using an Ultrospec 2100 Pro spectrophotometer (Biochrom, Cambridge, UK) at room temperature. Disposable UV cuvettes were used. The absorbance was measured in the wavelength range of 400–750 nm.

### 4.3. Dynamic Light Scattering Technique and Zeta Potential Measurements

Determination of particle size and its distribution was carried out using the dynamic light scattering (DLS) technique with a DynaPro NanoStar (Wyatt Technology LLC., Santa Barbara, CA, USA) device. The hydrodynamic radius measurement range for the DLS instrument spans from 0.2 to 1000 nm. Colloids were placed in disposable cuvettes, and measurements were taken in triplicate, with 10 acquisitions for each. The measurement parameters were as follows: laser wavelength of 663 nm, laser power of 100 mW, scattering angle of 90°, and temperature of 25 °C.

Zeta potential values were measured by dynamic light scattering using the Nano Zetasizer (Malvern Panalytical, Malvern, UK). Colloids were placed in disposable folded capillary zeta potential cells (Cat number DTS1070, Malvern Panalytical, United Kingdom) and analyzed in triplicate, with 20 acquisitions for each. The measurements were conducted under the following parameters: laser wavelength of 663 nm, laser power of 4 mW, scattering angle of 173°, and temperature of 25 °C. The stability of the produced complexes was investigated using UV-Vis spectroscopy, DLS technique, and zeta potential measurements over a period of 3 months.

### 4.4. Atomic Force Microscopy

The microscopy technique was used to investigate the morphology, such as shape, size, and the formation of agglomeration or aggregation. Atomic Force Microscopy (AFM) measurements were obtained using a MultiMode8-HR (Bruker, Billerica, MA, USA) microscope. AuNPs/PAMAM G2 colloids at concentrations of 0.5 mg/mL with a volume of 10 µL were placed onto silicon substrates and dried at room temperature. The images were acquired in tapping mode in air, using an RTESPA-300 cantilever (Bruker, USA). Scan parameters: size 5 × 5 µm at a resolution of 512 × 512 data points, with scan rates of 1 Hz.

### 4.5. Infra-Red Spectroscopy

Fourier Transform Infrared spectroscopy measurements were performed using a Nicolet iS50 FT-IR spectrometer (Thermo Scientific, Waltham, MA, USA) equipped with an attenuated total reflectance (ATR) accessory, operating at a resolution of 1 cm^−1^. Colloidal samples, at a concentration of 0.5 mg/mL with a volume of 50 L, were applied onto cleaved Si(001) wafers and left to dry under ambient conditions.

### 4.6. Cell Culture

Human breast adenocarcinoma MCF-7 cell line (cat. no. HTB-22^TM^) and human osteosarcoma Saos-2 cell line (cat. no. HTB-85) were obtained from ATCC (Manassas, VA, USA). Both cell lines were cultured under sterile conditions and passaged according to the manufacturer’s protocols. The following materials were sourced from ATCC (USA): McCoy’s 5A Medium, Dulbecco’s Modified Eagle Medium (DMEM), Fetal Bovine Serum (FBS), a Penicillin/Streptomycin cocktail, and a 0.25% (*w*/*v*) Trypsin–0.53 mM EDTA solution. Phosphate-buffered saline (PBS) was purchased from Gibco (Waltham, MA, USA).

MCF-7 breast cancer cells were routinely cultured in complete DMEM supplemented with 10% FBS. Saos-2 cells were cultured in McCoy’s 5A medium with L-glutamine, containing 10% FBS. Cells were maintained in 75 cm^2^ culture flasks in a humidified incubator at 37 °C with 5% CO_2_. For both cell lines, subculturing was performed every 3–4 days, coinciding with the appearance of the first floating cells in the medium and achieving approximately 90% confluence.

### 4.7. Cytotoxicity Measurements (XTT Assay)

The tetrazolium salt XTT (2,3-bis[2-methoxy-4-nitro-5-sulfophenyl]-2H-tetrazolium-5-carboxanilide) was purchased from Biological Industries (Cromwell, CT, USA). Depending on the cell line, the seeding density on a 96-well plate was 20,000 per well for MCF-7 and 10,000 for Saos-2, respectively. The AuNPs/PAMAM G2 colloids were centrifuged at 12,000× *g* for 30 min before being added to the cell cultures. The resulting pellet was washed twice and resuspended in serum-free culture medium and gold nanoparticle concentrations were prepared using serial dilutions. The initial concentration of 1:4 AuNPs/PAMAM G2 colloids, from which subsequent dilutions were prepared, was 0.5 mg/mL.

After 24 h of MCF-7 cell culture, the culture medium was replaced with suspensions of AuNPs/PAMAM complexes at final concentrations of 1, 2.5, 5, 10, 15, 20, 25, and 40 μg/mL in serum-free medium. Since the AuNPs/PAMAM complexes exhibited stronger toxicity in the Saos-2 cell line, the following concentrations were chosen for testing: 1, 2.5, 3.5, 5, 7.5, 10 and 15 μg/mL. Also, for the Saos-2 cell line, gold nanoparticles for culturing were administered in serum-free medium. Control cells received serum-free medium without nanoparticles. Following an additional 24 h incubation, cell viability was assessed using the XTT assay. The absorbance at 450 nm was measured for each well using a Victor X4 microplate reader (Perkin Elmer, Waltham, MA, USA). Cell viability was expressed as a percentage of the negative control (NC) cells, i.e., the untreated cells. Statistical analyses were performed using OriginPro 2017 software. One-way analysis of variance (ANOVA) was applied to assess differences between groups. Statistical significance was set at * *p* < 0.01 and ** *p* < 0.001. Additionally, EC_10_, EC_25_, and EC_50_ concentrations of AuNPs/PAMAM G2 were calculated, which correspond to 90, 75 and 50% of cellular viability. Data on the cellular viability are presented as a mean ± standard error of the mean.

## 5. Conclusions

The search for novel drug delivery systems and contrast agents for computed tomography continues to pose significant challenges to researchers. Polyamidoamine dendrimers, known for their high degree of surface functionality, have been widely explored in nanomedicine research; offer a wide range of applications. Our work presents a contemporary approach to utilizing PAMAM dendrimers as stabilizers in the synthesis of gold nanoparticles. The obtained conclusions are outlined in the following points:

- Three synthesis methods were employed to produce gold nanoparticles stabilized with PAMAM G2 dendrimers. All three types of synthesis methods are repeatable. Both sonication and unassisted methods enable the large-scale production of nanoparticles.
- The obtained nanoparticles synthesized using the second generation of PAMAM dendrimers exhibit long-term colloidal stability for up to 3 months.
- Among the tested formulations, the colloid PAMAM G2:HAuCl_4_ ratios of 1:4 were selected for in vitro cytotoxicity evaluation. Nanoparticles synthesized via sonication exhibited the lowest cytotoxicity compared to those produced using microwaves or without assistance.
- These studies provide a foundation for further investigations into the mechanisms underlying the biological activity of PAMAM-stabilized gold nanoparticles and their potential use in cancer treatment.

The interaction between nanoparticles and human cells has been a focus for many groups over the past decade. Nanotechnology and its associated nanomaterials offer the potential to exploit the unique properties of supramolecular assemblies and nanomaterials, making previously inaccessible effects available for new applications. Maintaining the stability of nanoparticle colloids is crucial for their subsequent application as drug carriers. Long-term stability enables a controlled and sustained release of active substances, reducing the frequency of drug administration and thereby making therapy less burdensome for patients.

## Figures and Tables

**Table 1:** The hydrodynamic diameter values for the AuNPs/PAMAM colloids (sonication synthesis) over time, along with the percentage of mass.

Sample–Sonication	Population	Hydrodynamic Diameter During Time [nm] (%Mass)			
		24 h	1 Week	1 Month	3 Months
1:2	Peak 1	3.6 (99.7%)	2.6 (98.8%)	2.6 (98.9%)	2.0 (98.8%)
	Peak 2	42.8 (0.3%)	48.6 (1.2%)	26.0 (1.1%)	24.6 (1.2%)
1:3	Peak 1	7.6 (97.2%)	5.2 (99.5%)	4.8 (98.3%)	3.6 (98.6%)
	Peak 2	33.0 (2.7%)	42.2 (0.5%)	32.4 (1.7%)	26.0 (1.4%)
	Peak 3	82.2 (0.2%)	-	-	-
1:4	Peak 1	19.2 (100%)	20.2 (100%)	18.8 (100%)	18.4 (100%)
1:5	Peak 1	2.6 (93.0%)	3.6 (86.3%)	4.8 (79.4%)	6.4 (99.2%)
	Peak 2	19.0 (6.4%)	16.2 (12.8%)	13.6 (18.7%)	-
	Peak 3	128.2 (0.6%)	111.6 (0.9%)	102.2 (93.9%)	77.6 (0.8%)

**Table 2:** The hydrodynamic diameter values for the AuNPs/PAMAM colloids (microwave synthesis) over time, along with the percentage of mass.

Sample–Microwave	Population	Hydrodynamic Diameter During Time [nm] (%Mass)			
		24 h	1 Week	1 Month	3 Months
1:2	Peak 1	*immeasurable*	*immeasurable*	1.8 (99.3%)	30.2 (100%)
	Peak 2			20.1 (0.7%)	-
1:3	Peak 1	*immeasurable*	1.2 (98.6%)	-	-
	Peak 2		14.4 (1.4%)	20.4 (100%)	24.2 (100%)
1:4	Peak 1	9.6 (94.5%)	7.0 (98.7%)	6.6 (99.0%)	8.6 (97.9%)
	Peak 2	102.4 (5.5%)	103 (1.3%)	104.4 (1.0%)	114.6 (2.1%)
1:5	Peak 1	5.2 (96.7%)	4.4 (93.0%)	10.0 (81.8%)	4.2 (97.6%)
	Peak 2	34.2 (0.8%)	38.2 (3.2%)	30.4 (2.1%)	17.8 (1.7%)
	Peak 3	157.4 (2.5%)	173.2 (3.7%)	175.4 (16.2%)	152.4 (0.8%)

**Table 3:** The hydrodynamic diameter values for the AuNPs/PAMAM colloids (unassisted synthesis) over time, along with the percentage of mass.

Sample–Unassisted	Population	Hydrodynamic Diameter During Time [nm] (%Mass)			
		24 h	1 Week	1 Month	3 Months
1:2	Peak 1	*immeasurable*	*immeasurable*	20.2 (100%)	27.2 (100%)
1:3	Peak 1	*immeasurable*	15.2 (99.9%)	21.0 (100%)	22.8 (100%)
	Peak 2		213.4 (0.1%)	-	-
1:4	Peak 1	1.2 (98.5%)	-	-	-
	Peak 2	14.0 (1.5%)	15.6 (100%)	17.2 (100%)	16.6 (100%)
1:5	Peak 1	7.8 (92.5%)	7.6 (92.4%)	9.0 (91.2%)	9.2 (94.4%)
	Peak 2	-	21.6 (4.7%)	40.2 (2.8%)	-
	Peak 3	163.4 (7.5%)	150.0 (2.9%)	150.8 (6.0%)	143.6 (5.6%)

**Table 4:** Summary of cell viability values for MCF-7 cells: EC_10_, EC_25_ and EC_50_ correspond to the 90, 75, and 50% of the control cellular viability. Data are presented as a mean ± standard error of the mean.

	Sonication [µg/mL]	Microwave [µg/mL]	Unassisted [µg/mL]
EC_10_	3.2 ± 0.7	3.3 ± 0.7	5.5 ± 0.7
EC_25_	13.1± 0.3	9.3 ± 0.3	11.7 ± 0.4
EC_50_	25.4 ± 0.4	20 ± 0.7	22.2 ± 0.2

**Table 5:** Summary of cell viability values for Saos-2 cells: EC_10_, EC_25_ and EC_50_ correspond to the 90, 75, and 50% of the control cellular viability. Data are presented as a mean ± standard error of the mean.

	Sonication [µg/mL]	Microwave [µg/mL]	Unassisted [µg/mL]
EC_10_	2.3 ± 0.2	1.4 ± 0.1	1.7 ± 0.3
EC_25_	3.9 ± 0.2	2.9 ± 0.1	3.2 ± 0.2
EC_50_	6.6 ± 0.1	5.4 ± 0.1	5.7 ± 0.1

## Data Availability

The original contributions presented in this study are included in the article. Further inquiries can be directed to the corresponding author.

## Abbreviations

The following abbreviations are used in this manuscript:

ATR	attenuated total reflectance
AuNPs	Gold nanoparticles
CT	Computed Tomography
DLS	Dynamic Light Scattering
DMEM	Dulbecco’s Modified Eagle Medium
EC	effective concentration
FBS	Fetal Bovine Serum
FT-IR	Fourier transform infrared spectroscopy
HAuCl_4_	chloroauric acid (hydrogen tetrachloroaurate)
MCF-7	human breast adenocarcinoma cell line
NC	negative control
PAMAM	polyamidoamine
PBS	Phosphate-buffered saline
Saos-2	human osteosarcoma cell line
UV-Vis	ultraviolet-visible spectroscopy
XTT	2,3-bis[2-methoxy-4-nitro-5-sulfophenyl]-2H-tetrazolium-5-carboxanilide
